# Histone acetylation modifications: A potential targets for the diagnosis and treatment of papillary thyroid cancer

**DOI:** 10.3389/fonc.2022.1053618

**Published:** 2022-11-29

**Authors:** Chongyang Chen, Jingfang Liu

**Affiliations:** ^1^ The First Clinical Medical College, Lanzhou University, Lanzhou, Gansu, China; ^2^ Department of Endocrinology, the First Hospital of Lanzhou University, Lanzhou, Gansu, China

**Keywords:** papillary thyroid cancer, histone, acetylation, deacetylation, epigenetic

## Abstract

Thyroid cancer is a common malignancy of the endocrine system, with papillary thyroid cancer (PTC) being the most common type of pathology. The incidence of PTC is increasing every year. Histone acetylation modification is an important part of epigenetics, regulating histone acetylation levels through histone acetylases and histone deacetylases, which alters the proliferation and differentiation of PTC cells and affects the treatment and prognosis of PTC patients. Histone deacetylase inhibitors induce histone acetylation, resulting in the relaxation of chromatin structure and activation of gene transcription, thereby promoting differentiation, apoptosis, and growth arrest of PTC cells.

## Introduction

Thyroid cancer (TC) accounts for approximately 95.01% of all malignant neoplasms of the endocrine system, with men and women accounting for approximately 24.05% and 75.95% of all newly diagnosed TC, respectively (1). Approximately 2.10% of all new malignancies diagnosed in China are TC, and it is the most common cancer diagnosed among women under 30 years of age in China (2). There are three main histological types of TC (3): differentiated thyroid cancer (DTC), which includes papillary thyroid cancer (PTC) and follicular thyroid cancer (FTC); and undifferentiated thyroid cancer (UTC), which includes poorly differentiated thyroid cancer (PDTC) and anaplastic thyroid cancer (ATC); and the last type is medullary thyroid cancer (MTC). PTC is the most common pathological type, accounting for approximately 85% of TCs (4, 5), and an increase in newly diagnosed cases of PTC has led to a threefold increase in the prevalence of TC over the past 30 years (4).

Patients with well-differentiated TC have shown good responses to treatment and favorable prognoses after surgical resection and radioactive iodine treatment combined with thyroid hormone suppressive therapy (6). However, some genes associated with aggressive clinical features are aberrantly expressed in some cases of PTC. Indeed, the downregulation of the paired box 8 (*PAX8*) and peroxisome proliferator-activated receptor gamma (*PPARG*) genes in PTC tissues leads to the metastasis of cancer cells to lymph nodes and distant tissues. Additionally, some PTC patients with these mutations put up dedifferentiated appearances, such as radioiodine refractory (7, 8), which can result in poor prognosis. As the incidence of PTC is increasing every year and its aggressive features can lead to a poor prognosis, early identification and diagnosis of PTC would be beneficial to allow for better treatment of the disease.

At present, the diagnosis of TC mainly relies on ultrasound-guided fine-needle aspiration biopsies, but the method is invasive and carries certain risks. In addition, it can be difficult to identify benign and malignant tumors using imaging examinations. In recent years, several molecular biomarkers have emerged as effective tools for early diagnosis and therapeutic monitoring of TCs (9). Acetyl-CoA carboxylase beta, cyclin D1, B cell lymphoma 2 (BCL2), PAX8, and PPARG have been reported to play important roles in the pathogenesis of PTC (7, 10). Mutations in genes such as vraf murine sarcoma viral oncogene homolog B1 (*BRAF ^V600E^*) and the Telomerase reverse transcriptase (*TERT*) promoter (9, 11), as well as epigenetic modifications such as specific non-coding RNA and DNA methylation modifications (12), are useful diagnostic and prognostic markers of PTC. Methylation, acetylation, phosphorylation, and ubiquitination modifications to histones may be involved in the development of TC. Studies have shown that histone acetylation and deacetylation modifications are closely related to the proliferation, differentiation, and growth of PTC (13). This paper reviews the latest research on histone acetylation and deacetylation modifications in PTC to propose novel targets for the clinical diagnosis and treatment of PTC, as well as explaining the known molecular biological mechanisms of PTC.

## Histone acetylation modifications

Histones are divided into five families: H1, H2A, H2B, H3, and H4. With two molecules of H2A, H2B, H3, and H4, respectively, join to form a histone octamer complex, which binds to a 147 bp double-stranded DNA helix for 1.75 coils. H1 then binds to the complex to form a nucleosome, which is the basic structural unit of chromatin. Each histone has multiple evolutionarily conserved N-terminal tails extending out of nucleosomes, which have been detected *in vitro* and are targets of many signaling pathways involved in post-transcriptional modifications (14). These post-transcriptional modifications are involved in the expression of many biological traits that are not genetically involved.

**Figure 1:**
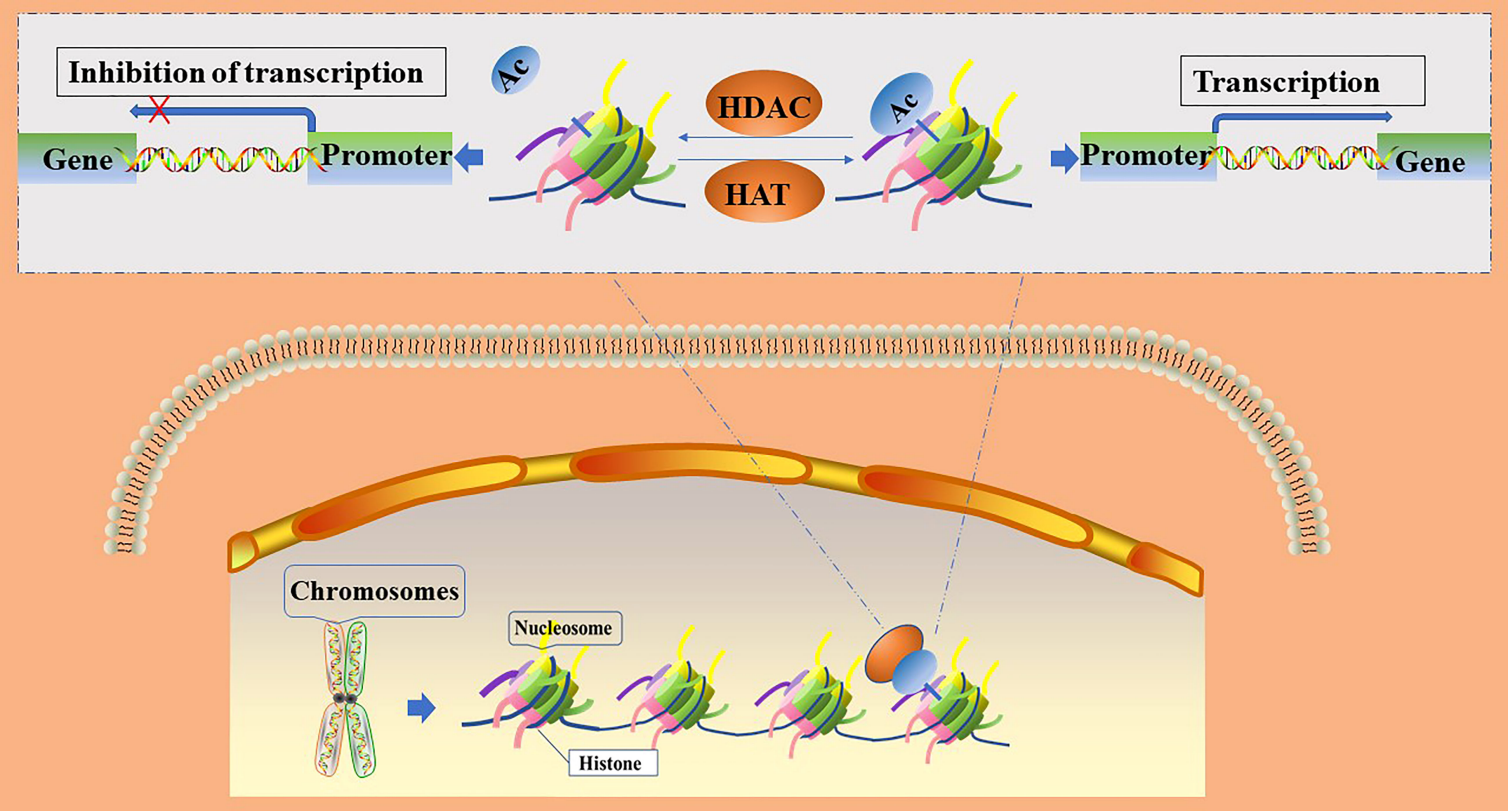
Regulation of gene transcription by histone acetylation and deacetylation. Ac, acetylation; HATs, histone acetylase; HDACs, histone deacetylase.

The acetylation of histones is an important mechanism of epigenetic transcriptional regulation, which can alter the structure of chromatin, thereby affecting the transcription of genes (15). Within the eukaryotic nucleus, histone acetylation and deacetylation are in dynamic equilibrium and are jointly regulated by histone acetyltransferases (HATs) and histone deacetylases (HDACs), respectively. HATs transfer acetyl groups to the ε-amino groups of histone lysine residues for participation in post-translational modifications (16); histone acetylation sites mainly occur at the following lysine residue positions: 9, 14, 18, and 27 of histone H3 or 5, 8, 12, and 16 of histone H4 (16). After histone acetylation, lysine residues located at the N-terminus have negative charges, which repel negatively charged DNA. This leads to the relaxation of the chromatin structure and opens up the binding sites for transcription factors in the gene promoter region. Transcription factors are then able to bind easily to the corresponding sites and promote gene expression, whereas histone deacetylation leads to the silencing of genes by blocking the access of transcription factors to gene promoter binding sites (16, 17). (**Figure 1**) The level of histone acetylation plays an important role in the development of many tumor types, including breast, colon, lung, liver, pancreatic, prostate, and thyroid cancers (18). Increased histone deacetylation resulting from the overexpression of HDACs suppresses the transcription of tumor suppressor genes and leads to unrestricted tumor proliferation, evasion of apoptosis, and rapid cell cycle progression, thereby promoting tumorigenesis. Therefore, the acetylation level of histones is considered a biomarker for evaluating the prognosis of many tumors (19).

## Histone acetylation modifications and papillary thyroid cancer

HATs are divided into two families according to their homology and functional role. The MYST family contains a highly conserved MYST domain composed of zinc-finger and acetyl-CoA binding, and includes the males-absent on the first protein (MOF; also called Lysine acetyltransferase 8, KAT8) and 60 kDa Tat-interactive protein (Tip60; also called Lysine acetyltransferase 5, KAT5) HATs, etc. (20). The general control of amino acid synthesis protein 5 (GCN5) related N-acetyltransferase (GNAT) superfamily contains bromodomains (BRDs) and includes the p300/CREB -associated factor (PCAF) and GCN5 HATs, etc. (21).

**Figure 2:**
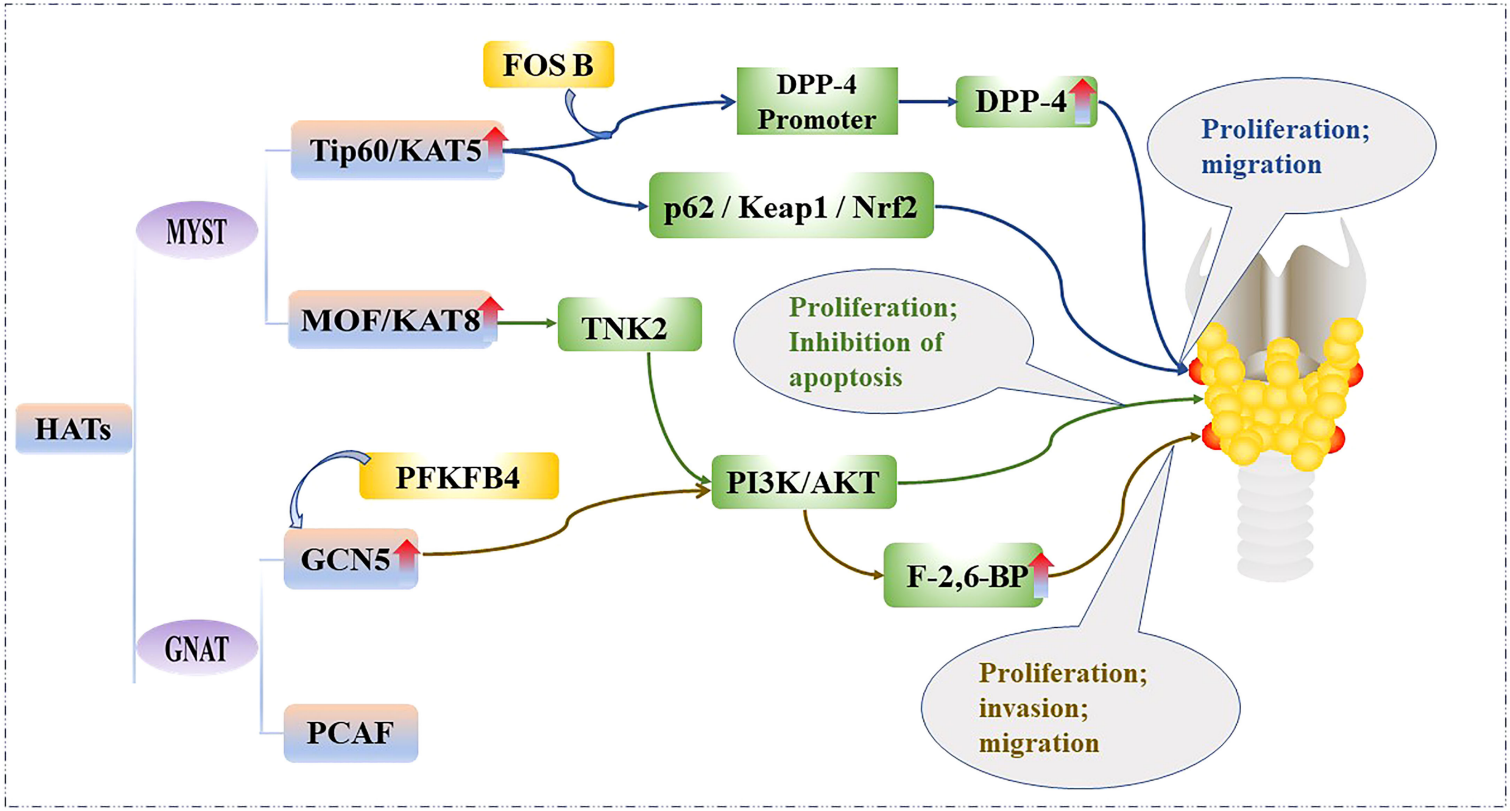
Mechanistic pathways of histone acetylation in papillary thyroid cancer. HATs, Histone acetyltransferases; Tip60, 60 kDa Tat-interactive protein; KAT5, Lysine acetyltransferase5; FOSB, FBJ murine osteoscarcoma viral oncogene homolog B; DPP-4, Dipeptidyl peptiase IV; p62/Keap1/Nrf2, Sequestosome 1/Kelch-like ECH-associated protein 1/NF-E2-related factor 2; MOF, Males-absent on the first protein; KAT8, Lysine acetyltransferase 8; TNK2, Tyrosine kinase 2; PI3K/AKT, phosphatidylinositol 3-kinase/protein kinase B; GCN5, General control of amino acid synthesis protein 5; GNAT, GCN5-related N-acetyltransferase superfamily; PCAF, p300/CBP associated factor; PFKFB4, 6-phosphofructo-2-kinase; F-2,6-BP, Fructose-2,6-bisphosphatase.

HAT levels are generally elevated in PTC tissues and cell lines, and bind to the promoter regions of some genes to acetylate them and promote their transcription. Therefore, HATs can promote the proliferation and metabolism of PTC cells. For example, Tip60 is recruited by FBJ murine osteoscarcoma viral oncogene homolog B (FOSB) to acetylate the promoter histone of dipeptidyl peptidase IV (DPP4), which increases DPP4 transcription, as well as activates p62/KEAP1/NRF2 signal transduction, thereby promoting the growth of PTC cell lines: IHH4, CUTC5, and TPC1 (22). In addition, MOF catalyzes the acetylation of lysine at position 16 of histone H4, which is associated with a variety of biological processes, including gene transcription, cell cycle, early embryonic development, and tumorigenesis (23). MOF is significantly upregulated in most PTC tissue samples and cell lines; it binds to the promoter of Tyrosine kinase 2 (TNK2) to initiate its transcription and subsequently increases the phosphorylation of protein kinase B, which activates the phosphatidylinositol 3-kinase/protein kinase B (PI3K/AKT) pathway, and ultimately promotes the proliferation of PTC cell lines BHP10-3 and IHH4 (24). Furthermore, 6-phosphofructo-2-kinase (PFKFB4) knockdown has been shown to inhibit PTC tumorigenesis mediated by GCN5. PFKFB4 promotes the expression of fructose-2,6-bisphosphatase (F-2,6-BP), a key enzyme in glycolysis, through upregulation of GCN5 and PI3K/AKT signaling pathways, thereby eliminating the inhibitory effect on PTC cell growth and promoting the invasion, proliferation and migration of PTC cell lines IHH4 and TPC1 (25). The effect of the HATs on the PTC is shown in **Figure 2**.

High levels of HATs lead to increased acetylation levels in PTC cells. Detection of acetylation levels in thyroid tissue can help distinguish normal from early-stage carcinoma tissue and poorly differentiated tissue. Studies have shown that follicular adenoma, PTC, FTC, and undifferentiated carcinoma tissues have higher levels of acetylation of lysine residues 9–14 in histone H3 than in normal thyroid tissue. In addition, undifferentiated TC has lower levels of acetylated lysine residue 18 in H3 than in differentiated TC, and acetylation of lysine residue 12 in H4 levels is significantly higher in adenomas than in normal tissues (26). PTC and benign thyroid nodule samples can be divided into two groups based on differences in genome-wide acetylation levels of histone H3 residue 27. For instance, at PTC-specific genomic sites, the histone H3 residue 27 acetylation signals were remarkably lower in benign thyroid nodules than in the corresponding locus in PTC tissue (13). The activation of some genes or altered hormone levels in PTC is associated with histone acetylation levels. For example, expression of the rearranged during transfection (*RET*) and *BRAF* oncogenes increases the levels of H3K9-K14Ac and H3K18Ac in rat PTC cell lines; thyroid-stimulating hormone (TSH) increases the levels of H3K18Ac and decreases the levels of H3K9-K14Ac and H4K12Ac in PTC cell lines (26).

Histone acetylases can recognize lysine residues through BRDs (27). Thus, blocking BRDs can inhibit proliferation and promote differentiation of TC tissue. For example, the BRD and extra-terminal (BET) families of proteins recognize lysine residues at positions 5, 8, and 12 of histone H4 (28). This includes four proteins: BRD2, BRD3, BRD4, and BRDT. BRD4 is an important member of the BET family that initiate the transcription of downstream genes by binding to acetylated histones (29). The BET inhibitor, JQ1, is a novel epigenetic anticancer drug that can effectively reduce cell viability and induce cell death by binding to and inhibiting the acetyl lysine recognition sites of BRD4. JQ1 recruits transcription elongation factor complexes to acetylated chromatin regions and promotes transcription through interaction with RNA polymerase I (28). As expected, BRD4 expression levels are upregulated in PTC tissues and in BCPAP and K1 PTC cell lines resulting in cancer cell growth. JQ1 inhibition of BRD4 leads to cell cycle arrest in the G0/G1 phase in PTC cells, enhanced ^131^I uptake *in vitro*, and tumor growth inhibition *in vivo* (30).

The SWI/SNF complex is able to maintain TC cell differentiation by targeting specific enhancers and regulating lysine acetylation at position 27 of histone H3, deletion of SWI/SNF complex subunits leads to chromatin suppression in *BRAF^V600E^* mutant PTC cell lines, further reducing DNA binding site accessibility to thyroid-specific genes due to *BRAF^V600E^* mutations and increasing resistance to redifferentiation therapies with MAPK inhibitors (31).

## Histone deacetylation and papillary thyroid cancer

HDACs can be divided into four classes (32, 33): class I includes HDAC1–HDAC3 and HDAC8; class II can be divided into class IIa (HDAC4, HDAC5, HDAC7, and HDAC9) and class IIb (HDAC6 and HDAC10); class III includes the nicotinamide adenine dinucleotide-dependent deacetylase and sirtuins (SIRTs), including SIRT1–SIRT7; and class IV contains HDAC11. HDACs remove acetyl groups from histone lysine residues and tighten the chromatin structure by interacting with positively charged histone N-terminus residues with negatively charged DNA, thereby deactivating transcription by preventing the gene promoter region from binding to transcription factors (33). In addition, HDACs also bind to and deacetylate non-histone proteins, such as the nuclear factor kappa light chain enhancer of activated B cells (NF-κB) (34, 35) and tumor suppressor, p53 (36).

**Figure 3:**
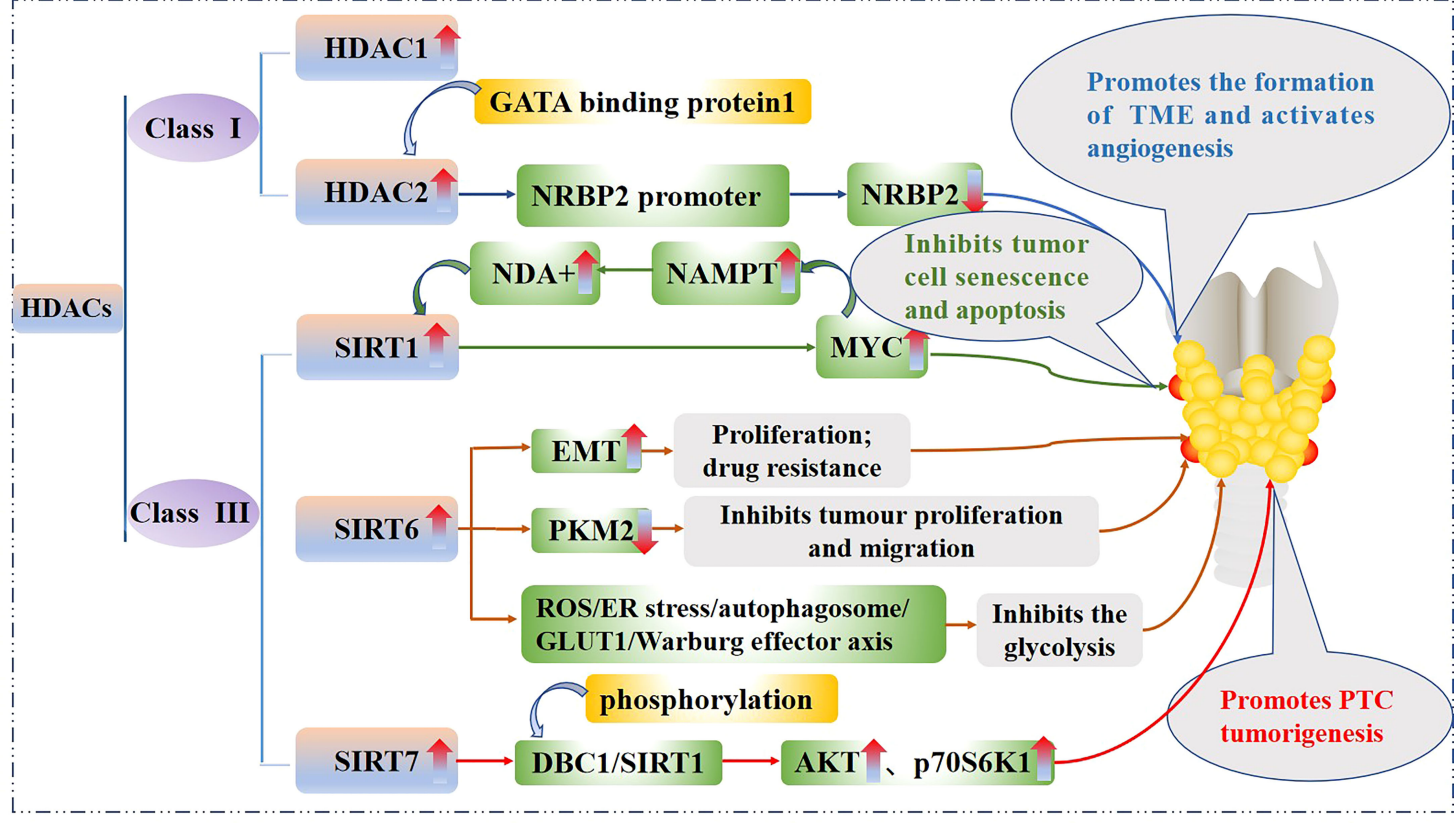
Mechanistic pathways of histone acetylation in papillary thyroid cancer. HDAC, Histone deacetyltransferases; NRBP2, Nuclear receptor binding protein 2; SIRT, Sirtuins; NAMPT, Nicotinamide-phosphoribosyltransferase; NAD+, Nicickamide adenine dinucleotide; MYC, Myelocytomatosis oncogene; EMT, Epithelial mesenchymal transition; PKM2, Pyruvate kinase M2; ROS, Active oxygen; ER, endoplasmic reticulum; GLUT1, Glucose transporter 1; DBC1, Promoter of deleted in breast cancer-1; AKT, protein kinase B.

Some HDACs are aberrantly expressed in PTC and this affects tumorigenesis, cancer cell proliferation, and energy metabolism. The effect of the HDACs on the PTC is shown in **Figure 3**. In fact, HDAC1 and HDAC2 expression levels are mildly increased in PTC tissues relative to normal tissues (37), Nuclear receptor binding protein 2 (NRBP2) reduces TC tumorigenesis and M2 macrophage infiltration *in vivo*, GATA binding protein 1 recruits HDAC2 to the NRBP2 promoter region, inhibits NRBP2 expression by reducing H3K9ac levels, blocks NRBP2 inhibition in PTC histiocytes and BCPAP and TPC-1 cell lines, and thereby promotes the formation of tumor microenvironment(TME) and activates angiogenesis (38). A META analysis showed that SIRT1 overexpression was detrimental to the overall survival of patients with solid malignancies (39), and SIRT1 nuclear staining was approximately 2.5-fold higher in PTC tissues than in normal tissues. In addition, the levels of SIRT1 selectively and positively correlated with myelocytomatosis oncogene (*MYC*) protein levels in PTCs (40), and this was shown to be through the lysine-specific deacetylation of MYC by SIRT1, which led to the subsequent increase in transcriptional activity of MYC. Furthermore, MYC increases nicotinamide adenine dinucleotide (NAD+) by increasing the expression of nicotinamide phosphoribosyltransferase (NAMPT), which in turn enhances SIRT1 activity, and this positive feedback inhibits tumor cell senescence and apoptosis, maintaining the development and progression of PTC (41).

SIRT6 binds to and mediates the deacetylation of lysine residue 433 of pyruvate kinase M2 (PKM2), resulting in the elimination of the nucleoprotein kinase and transcriptional coactivator functions of the latter, which in turn reduces HepG2 cells proliferation, migration potential, and invasiveness (42). In addition, SIRT6 not only promotes the aggressive phenotype and epithelial mesenchymal transition(EMT) of PTC cell lines TPC1 and BCPAP (43), thus increasing its proliferation and drug resistance, but also increases the production of reactive oxygen species (ROS), then activates endoplasmic reticulum (ER) stress and inhibits glucose transporter 1 (GLUT1) through autophagosome-mediated degradation, thereby suppressing the glycolysis in PTC cell lines TPC1 and K1 *via* the SIRT6/ROS/ER stress/autophagosome/GLUT1/Warburg effector axis (44, 45).

SIRT7 highly selectively removes the acetyl group of the lysine residue at position 18 of histone H3. This function of SIRT7 is associated with oncogenic transformation, tumor aggressiveness, and poor prognosis in patients. Thus SIRT7 plays a key role in chromatin regulation, cellular transformation and tumorigenesis (46). SIRT7 expression is increased in PTC tissues as well as in the PTC cell lines FTC133, 8505C, TPC1, BCPAP, IHH4 and K1, in addition, SIRT7 knockdown remarkably inhibited proliferation, colony formation, migration and invasion, and also induced cycle arrest and apoptosis in PTC cancer cells, SIRT7 promotes thyroid tumorigenesis through phosphorylation of the promoter of deleted in breast cancer 1 (DBC1)/SIRT1 axis and activation of AKT and p70S6K1 in BALB/c lymphatic-free mice (47).

The development of PTC is closely linked to histone acetylation or deacetylation, which may lead to a radioiodine-refractory phenotype or increased aggressiveness of PTC, or vice versa. Therefore, the early detection of invasive PTC and the appropriate clinical management strategies are facilitated by detecting changes in HATs and HDACs and the gene, transcriptional and translational levels associated with them.

## Effect of histone deacetylase inhibitors on thyroid cancer

Histone deacetylase inhibitors (HDACi) inhibit the activity of HDACs and cause unrestricted activity of HATs, which in turn induces the acetylation of histones resulting in the relaxation of chromatin structure and transcriptional activation. This leads to cell differentiation, apoptosis, and growth arrest (48, 49). HDACi include hydroxyl oxime acids (e.g., vorinostat, belinostat, and pabista), short-chain fatty acids (e.g., valproic acid, butyric acid, and phenylbutyric acid), benzamide (e.g., entinostat), cyclic tetrapeptides (e.g., romidepsin), sirtuin inhibitors (e.g., nicotinamide), and the specific class I/II HDAC inhibitor trichostatin A (TSA) (50). Almost all HDACi have cytostatic and cytotoxic effects on TC cells (48). HDACi can amplify therapeutic targets by increasing the expression of target genes and promoting tumor differentiation. For example, romidepsin induces thyroglobulin and sodium/iodide symporter (NIS) in TC tissues, thereby increasing the uptake of radioactive iodine. This promotes the sensitivity of TC tissue to radioactive iodine (51). HDACi can induce the activation of apoptosis-related proteins, such as caspases, and downregulate proliferation-related proteins, such as BCL2, leading to the death of TC cells (52). In addition, HDACi increases DNA damage induced by acetylation, leading to acute alterations in proliferative signaling (53) and apoptosis of TC cells.

Belinostat, vorinostat, romidepsin, pabisterostat, and valproic acid (VPA) can successfully induce growth arrest and apoptosis in TC cells (48). Belinostat causes antitumor effects by inhibiting the PI3K/AKT signaling pathway in TC cells (54), whereas TSA promotes TC cell differentiation by upregulating the expression of thyroid-specific genes associated with iodide handling (55). Romidepsin inhibits cell growth in ATC and PTC cell lines by up-regulating small G proteins (56). Pabisterostat dose-dependently induces histone acetylation and apoptosis-related protein expression in the SW576 TC cell line (57). VPA causes an increase in acetylated histones, thereby restoring the expression of dormant tumor suppressor genes and other genes associated with tumor cell differentiation, cell cycle arrest, and apoptosis. VPA in combination with the RAS inhibitor farnesyl thiosalicylic acid (salirasib) inhibits the proliferation of TC cells both *in vivo* and *in vitro*, synergistically reducing the proliferation of the TC cell line ARO (58).

A recent review outlines the progress of HDACi research in the treatment of PTC. In preclinical trials, most HDACi showed inhibitory effects on the growth and proliferation of PTC cell lines, and some HDACi reversed radioiodine tolerance, but in clinical trials, the efficacy of HDACi alone did not meet expectations (59). The results of a phase II clinical trial showed that 19 patients with TC treated with vorinostat (16 with DTC and 3 with MTC) did not achieve partial or complete remission (60). 13 patients with TC (11 with DTC and 2 with metastatic thyroid cancer) treated with panobinostat showed stable disease in 7 patients and progressive disease in the other 6 patients (61). Treatment of TC patients with romidepsin (8 patients with PTC, 2 with FTC and 11 Hürthle) resulted in stable disease in 13 patients, progressive disease in 7 patients and adverse events reported in 12 patients (62). In addition, 13 patients with TC (7 patients with PTC, 2 with follicular variant PTC, 2 with FTC and 2 with Hürthle) did not show increased radioiodine uptake after treatment with VPA and 6 patients showed progression (63).

Some genes related to iodine transport and thyroglobulin expression are central to the ability of TC to respond to radioiodine therapy (RAI). However, when genetic mutations occur (e.g. *BRAF*, *RAS* and *RET/PTC* rearrangements), they activate MAPK and PI3K/AKT signalling pathways, leading to reduced expression of iodine-handling genes in TC, particularly the *NIS* gene, disabling iodine uptake and resistance to RAI treatment (64). This change is associated with histone acetylation, for example, deacetylation of histone H3 and H4 lysine residues at the *NIS* promoter inhibits its expression in PTC patients with mutations in *BRAF^V600E^ (* 65). Redifferentiation therapy is the reprogramming of the cancer differentiation state and increasing the response of cancer cells to radioiodine therapy by promoting the expression of genes required for iodine uptake and thyroglobulin in TC cells. The kinase inhibitors targeting MAPK or PI3K pathways have shown promising results in redifferentiation therapy (66). Activation of MAPK signalling impairs the expression of thyroid-specific genes in TC, rendering them unresponsive to radioiodine treatment (64). CUDC-907 inhibits both HDAC and tyrosine kinase signalling pathways and significantly inhibits proliferation and migration, induces G2/M arrest and apoptosis in TC cell lines in preclinical studies (67).

The Notch signalling is associated with cell proliferation, differentiation, epithelial mesenchymal transition and angiogenesis, among other properties. differential expression of Notch may be associated with the histopathology and degree of cell differentiation of different subtypes of TC. Over-expression of Notch1 and Notch2 in PTC may increase the aggressiveness, but it has also been shown that Notch1 in PTC tissues and cells expression is extremely low. In addition, HDACi induces Notch signalling expression in TC cells in preclinical studies, but satisfactory results have still not been obtained in clinical trials (68).

In addition, studies have shown that the active ingredients of some herbs possess HDACi activity and have a significant inhibitory effect on PTC. For example, SIRT1 is a molecular target of resveratrol (RSV), which regulates the secretion of TSH and activates the MAPK signaling pathway to inhibit the growth of PTC and FTC (69). Triptolide (TPL), a biologically active diterpenoid isolated from plants, inhibits cell proliferation and induces apoptosis by downregulating NF-κB (70). TPL combination treatment with the novel heat shock protein inhibitor, BIIB021, induced cell death in ATC and PTC cell lines by inhibiting the PI3K/AKT signaling pathway and activating the DNA damage response (71). Evodiamine exerts its anticancer effects by inhibiting cell proliferation, invasion, and metastasis, while inducing apoptosis in many types of cancer cells (72). Evodiamine combined with vorinostat and troglitazone A modulates the production of Bcl2 family proteins, DNA damage response proteins, ROS, and AKT inactivation, which results in a decrease in acetylated protein levels and the viability of PTC cell lines TPC1 and SEL736 (73).

## Conclusion

TC is a common malignancy of the endocrine system, of which PTC is the most common pathological type. Recent studies have revealed that histone acetylation modifications play an important role in the development of PTC. HATs bind to the promoter regions of some genes related to cellular energy metabolism and growth and promote the transcription of these genes through acetylation, which facilitates the proliferation and growth of PTC cells. Increased acetylation of histones was observed in PTC tissues, with less acetylation detected in undifferentiated than in differentiated TCs. The expression of HDACs in PTC tissues and cells was upregulated, which reduced cellular senescence and apoptosis, and promoted an aggressive phenotype and epithelial mesenchymal transition. However, HDAC expression also inhibited the proliferation, migration, and invasion of PTC, and suppressed the glycolytic metabolism of tumor cells. The results are presented in **Table 1**. In preclinical trials, most HDACi showed inhibitory effects on the growth and proliferation of PTC cell lines, and some HDACi reversed radioiodine tolerance, but in clinical trials, the efficacy of HDACi alone did not meet expectations.

**Table 1:** Histone acetylase and histone deacetylase studies in PTC.

Enzymes	Category	Effects on PTC	References
	Tip60	FOSB increases DPP4 transcription by recruiting the histone acetyltransferase KAT5 and activates p62/Keap1/Nrf2 signaling to promote the growth and metastasis of PTC cells.	(22)
HATs	MOF	MOF activates the PI3K/AKT pathway by binding to the TNK2 promoter to promote thyroid cancer proliferation and inhibit apoptosis.	(24)
	GCN5	PFKFB4 promotes the expression of fructose-2,6-bisphosphatase, a key enzyme in glycolysis, through upregulation of GCN5 and PI3K/AKT signaling pathways, thereby eliminating the inhibitory effect on PTC cell growth and promoting the invasion, proliferation and migration of PTC.	(25)
	HDAC2	HDAC2 inhibits NRBP2 expression by reducing H3K9ac levels, and thereby promotes the formation of TME and activates angiogenesis.	(37, 38)
	SIRT1	SIRT1 increases MYC transcriptional activity through deacetylation, which in turn increases NAD+ through increased expression of NAMPT, thereby enhancing SIRT1 activity, and this positive feedback inhibits tumor cell senescence and apoptosis.	(41)
HDACs	SIRT6	Overexpression of SIRT6 promotes an aggressive phenotype and EMT in PTC; SIRT6 mediates acetylation of PKM2 and inhibits the proliferation and invasion of PTC cells; SIRT6 inhibits the glycolysis in PTC cells *via* the SIRT6/ROS/ER stress/autophagosome/GLUT1/Warburg effector axis.	(42–44)
	SIRT7	SIRT7 promotes thyroid tumorigenesis through DBC1/SIRT1 axis phosphorylation and activation of AKT and p70S6K1.	(47)

## Author contributions

All authors were involved in the conception of the manuscript, CC drafted the manuscript and JL revised it. All authors contributed to the article and approved the submitted version.

## Conflict of interest

The authors declare that the research was conducted in the absence of any commercial or financial relationships that could be construed as a potential conflict of interest.

## Publisher’s note

All claims expressed in this article are solely those of the authors and do not necessarily represent those of their affiliated organizations, or those of the publisher, the editors and the reviewers. Any product that may be evaluated in this article, or claim that may be made by its manufacturer, is not guaranteed or endorsed by the publisher.
